# Study on positive psychology from 1999 to 2021: A bibliometric analysis

**DOI:** 10.3389/fpsyg.2023.1101157

**Published:** 2023-03-01

**Authors:** Feifei Wang, Jia Guo, Guoyu Yang

**Affiliations:** ^1^Department of Developmental Psychology of Armyman, School of Psychology, Army Medical University, Chongqing, China; ^2^Department of Financial Management, Chongqing Business Vocational College, Chongqing, China

**Keywords:** positive psychology, developing trends, knowledge mapping, scientometrics, VOSviewer, CiteSpace

## Abstract

**Objective:**

Positive psychology is a revolution in the science of psychology as well as a new milestone in the development of human society. The purpose of the study was to use bibliometrics and visual analysis to assess the current state and trends in positive psychology research.

**Methods:**

The Web of Science Core Collection was searched for 4,378 papers on positive psychology between 1999 and 2021. The features of positive psychology research were analyzed using Microsoft Excel 2013, VOSviewer (1.6.17), and CiteSpace (5.8.R1).

**Results:**

The findings demonstrate a steady growth in positive psychology publications from 1999 to 2021. The United States (1,780) and Harvard University (104), respectively, were the most productive nations and organizations in this subject. *Frontiers in Psychology* was the most productive journal (288), while the *Journal of Personality and Social Psychology* had the most co-citations (8,469). Seligman was the most influential author, with 3,350 citations and 5,020 co-citations. The top ten co-cited references, in terms of citation explosion, suggesting that these papers provide the foundation for the growth of this discipline. The systematic review, character strengths, positive psychology intervention, language pleasure, and the COVID-19 pandemic are the focal points of research and development developments in this discipline.

**Conclusion:**

These findings have helped researchers in positive psychology find new ways to collaborate with partners, hot topics, and research frontiers.

## 1. Introduction

Positive psychology is a vibrant field of research, and concepts about the components of well-being predate the positive psychology movement. Diener (1984) evaluated the literature on subjective well-being (SWB) in 1984, then created and validated the Life Satisfaction Scale (SWLS) (Diener et al., 1985). Ryff (1989, 2014, 2022) has studied psychological well-being for over 30 years in an effort to determine its fundamental components, find what conditions encourage or hinder it, and investigate how it impacts health. In 1998, Seligman was elected president of the American Psychological Society. He advocated that psychologists and practitioners concentrate on enjoyment rather than illness reduction. Many psychologists advocate a greater emphasis on positive psychological development. It discusses how to develop attributes such as imagination, optimism, foresight, interpersonal talents, moral judgment, patience, humor, and fearlessness, as well as how to promote pleasure and life satisfaction (Gillham and Seligman, 1999). The Millennium issue of *American Psychologist* focuses on the emerging science of positive psychology. Psychologists are beginning to consider what advantages humans have at the end of the twentieth century. *Positive Psychology*, published in 2000 by Seligman and Csikszentmihalyi (2000), marked the formal beginning of positive psychology. So this manuscript will focus on the development of positive psychology after its formal beginning. Therefore, Seligman (2019) is often called the “Father of Positive Psychology.”

The application of psychological ideas, research, and intervention strategies to comprehend the good, adaptable, imaginative, and emotionally satisfying elements of human behavior is known as positive psychology (Seligman and Csikszentmihalyi, 2000). It shifts the research’s focus to “ordinary people” (Sheldon and King, 2001). Positive psychologists, like other natural or social scientists, seek to understand psychological structure, phenomena, and functions. There has been significant progress in the theory of positive psychology, as evidenced by PERMA (the five elements of well-being; Seligman, 2011), the dual-factor model of mental health (Suldo and Shaffer, 2008), the complete state model of health (Keyes, 2005), and the broaden-and-build theory of positive emotions (Fredrickson, 2001). Subsequently, positive psychology was widely used, including national psychological accounts of well-being (Diener and Seligman, 2018), positive psychotherapy (Seligman et al., 2006), a classification of strength and virtue (Snow, 2018), comprehensive soldier fitness (Lester et al., 2022), positive education (Seligman et al., 2009), and so on. With the advancement of positive psychology studies, positive psychology intervention has attracted researchers’ attention. Positive psychological intervention (PPI) is defined as “building its intervention on positive psychology theory and employing its coherent theoretical model to achieve the objective of promoting happiness” (Carr et al., 2020). Many positive psychology interventions have been shown to significantly boost well-being and minimize depressive symptoms (Sin and Lyubomirsky, 2009; Bolier et al., 2013; Carr et al., 2020). As the COVID-19 global health crisis unfolds, positive psychology is critical for sustaining mental wellness (Waters et al., 2021).

After more than two decades of development, many research papers have been published in the field of positive psychology research. Researchers systematically reviewed 1,336 articles published between 1999 and 2013 and found that positive psychology is a growing and dynamic subfield within the broader discipline of psychology (Donaldson et al., 2014). However, the field has grown rapidly in recent years, adding a large body of literature that requires us to use scientometric methods for analysis. Therefore, we must pay more attention to the research hotspots and trends in positive psychology. Scientometrics is a powerful tool for identifying emerging trends and hotspots in the subject (Chen, 2017; Hou et al., 2018). Bibliometric analysis is more objective and efficient than standard qualitative analysis approaches. In recent years, the advancement of scientific mapping techniques has increased (Cobo et al., 2011). Scientific mapping technologies generally capture a bibliographic record of a group of study fields and create an overview of the underlying knowledge domains. Typical tools are CiteSpace (Chen, 2006) and VOSviewer (van Eck and Waltman, 2010; Ding and Yang, 2020). Computationally aided literature reviews are not intended to replace expert-written reviews; rather, they are intended to provide an additional point of reference with some advantages (Chen et al., 2014b). Bibliometric analysis is currently used extensively in a variety of research areas, including depression (You et al., 2021; Zhou Y. et al., 2021); mindfulness research (Baminiwatta and Solangaarachchi, 2021); COVID-19 (Chen, 2020; Yu et al., 2020); and so on. The use of bibliometric analysis in clinical practice has become increasingly popular. Unlike typical expert-compiled evaluations, scientometrics covers a broader and more diverse range of critical issues. A collection of scholarly literature reflecting positive psychology research was used as input for this research. As a result, the purpose of this research is to conduct a bibliometric analysis of positive psychology research from 1999 to 2021 using the software packages CiteSpace and VOSviewer in order to better understand the field’s current condition, hotspots, and developmental trends.

## 2. Data and methods

### 2.1. Data acquisition

The study’s data source was the Web of Science database, which is the world’s most reliable citation database and has numerous high-quality papers (Huertas González-Serrano et al., 2019; Luo et al., 2021). The data is gathered mostly from the Web of Science Core Collection (WoSCC), which includes SCI-Expanded, SSCI, ESCI, and A&HCI. Due to the WoSCC literature retrieval database’s ongoing updating, we only conducted a single search on April 18, 2022.

The elements of the field of positive psychology were initially outlined by Seligman (2019) in 1998. Additionally, the APA Thesaurus included the index phrase “positive psychology” (Gallagher Tuleya, 2007) in June 2003. Positive psychology topics are also reflected by a variety of additional thesaurus phrases (for example, “well-being,” “life satisfaction,” “positive emotions,” “happiness,” and so on). It was challenging to establish the eligibility of the literature due to the abundance of terminology connected to the issue of positive psychology. This study focuses on the exact term “positive psychology” (Schui and Krampen, 2010), as well as the time period 1999–2021.

### 2.2. Inclusion criteria

The search yielded approximately 5,374 publications. The language utilized is English, and the type of literature is limited to “article” or “review.” This advanced search process excludes 996 articles. Finally, 4,378 publications were obtained and analyzed.

### 2.3. Analysis methods

Analytics used in the study include Microsoft Excel 2013; VOSviewer (1.6.17); and CiteSpace (5.8.R1). We use Microsoft Excel 2013 to analyze the changes and trends in the number of documents (Zhou T. et al., 2021). VOSviewer is a document analysis software package that has been developed by Van Eck and Waltman (van Eck and Waltman, 2010). It has been proven to have excellent visualization and analysis results and is widely used for document analysis (van Eck and Waltman, 2010; Liao et al., 2018). In this study, the software is used to analyze the features of positive psychology studies, such as Countries/Regions, institutions, journals, and authors. Using the method of full counting to construct a bibliometric network (Perianes-Rodriguez et al., 2016). The size of the nodes represents the number of publications, while the overall connection strength value illustrates the degree of collaboration between a subject and others (Zou and Sun, 2019).

CiteSpace is bibliometric analysis software (Chen, 2006), which can be used for the analysis of co-citations, burst detection, and emerging research trends in the literature (Chen et al., 2012; Hou et al., 2018). By developing a collection of visual knowledge maps, CiteSpace explores the states, hotspots, frontiers, and evolution processes in a scientific field.

The parameters of CiteSpace are as follows: time split between January 1999 and December 2021 (each slice is 1 year), the analysis items are selected as references, one node type is selected at a time, the selection criteria [g-index (*k* = 35)], and pruning (Pathfinder). A visual knowledge map is created using nodes and connections. In the map, each node represents one reference. The size of the nodes reveals the frequency of reference, while different colors of nodes stand for different years. In the center, the burst node as a red circle represents the number of co-occurrences or references that grow with time. Purple nodes represent the centrality and important knowledge exhibited by the data (Chen, 2012). The line of connection between nodes is taken to be a co-occurrence or co-cited relation; the thickness of the line signifies the strength of the relationship, and the color corresponds to the time of the first node (Liu and Chen, 2012). Cold to warm colors represent the early to recent. Betweenness centrality is another name for centrality. Nodes with high mental quality (>0.1) are frequently considered paradigmatic or pivotal moments in a discipline. An explosion of references to citations explores the trend and shows if the relevant writers have gotten significant attention on this subject (Chen et al., 2014a). Researchers may use this map to better understand new trends and identify hotspots by using burst detection and analysis (Chen et al., 2014a; Shi et al., 2022).

## 3. Results

### 3.1. Time trend analysis of publication outputs

**Figure 1:**
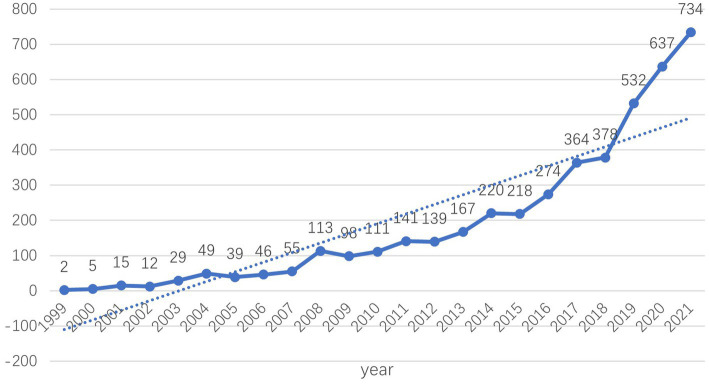
Trends in annual publications in positive psychology research.

The search yielded a total of 4,378 publications, of which 4,021 were articles and 357 were reviews. This annual publication may demonstrate the trend in this field of research, which we portray as a broken line chart (Figure 1). Figure 1 shows that the number of papers published between 1999 and 2021 continues to increase, indicating that related research areas are increasingly attracting academic interest. In particular, the literature on positive psychology has grown substantially in the last 3 years. The number of publications is expected to continue to grow.

### 3.2. Analysis of countries/regions

**Figure 2:**
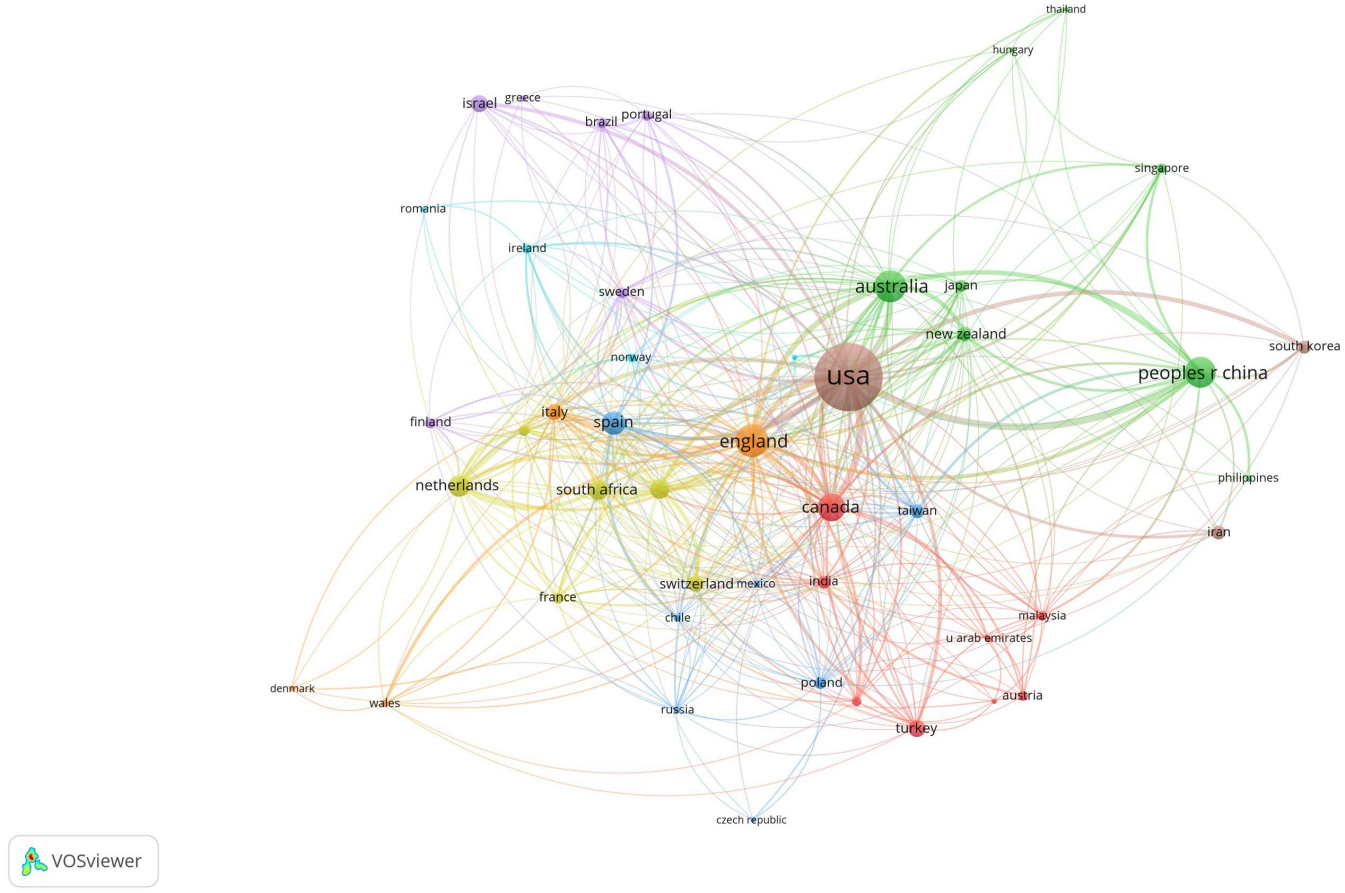
Map of countries/regions in positive psychology research.

Between 1999 and 2021, 96 countries/regions published research on positive psychology. We use the parameters of the number of publications (≥10) and the strength of the lines (≥1), generating 46 nodes and 428 links in the network of partner countries/regions (Figure 2). In Figure 2, we can see that the United States had the most publications (1,780), accounting for 40.66% (1,780/4,378), much outnumbering the rest, followed by England (420), Australia (388), the People’s Republic of China (361), and Canada (298). The table compares the top 10 countries/regions in terms of the number of publications, WoS citations, citations per study, and overall link strength (Table 1). The United States was the leading country, ranking first in terms of publications, WoS citations, citations per article, and total link strength, indicating that the United States is absolutely dominant in the field of positive psychology.

**Table 1:** Top 10 prolific countries/regions researching positive psychology.

Ranking	Publications	Citations WoS	Citations per paper	Total link strength	Country/region
1	1780	86,401	48.54	523	USA
2	420	14,218	33.85	294	England
3	388	9,990	25.75	248	Australia
4	361	4,467	12.37	185	Peoples R China
5	298	9,111	30.57	202	Canada
6	208	3,139	15.09	110	Spain
7	174	6,385	36.70	143	Netherlands
8	158	1,677	10.61	100	South Africa
9	136	5,125	37.68	143	Germany
10	107	1,917	17.92	35	Israel

### 3.3. Analysis of institutions

**Figure 3:**
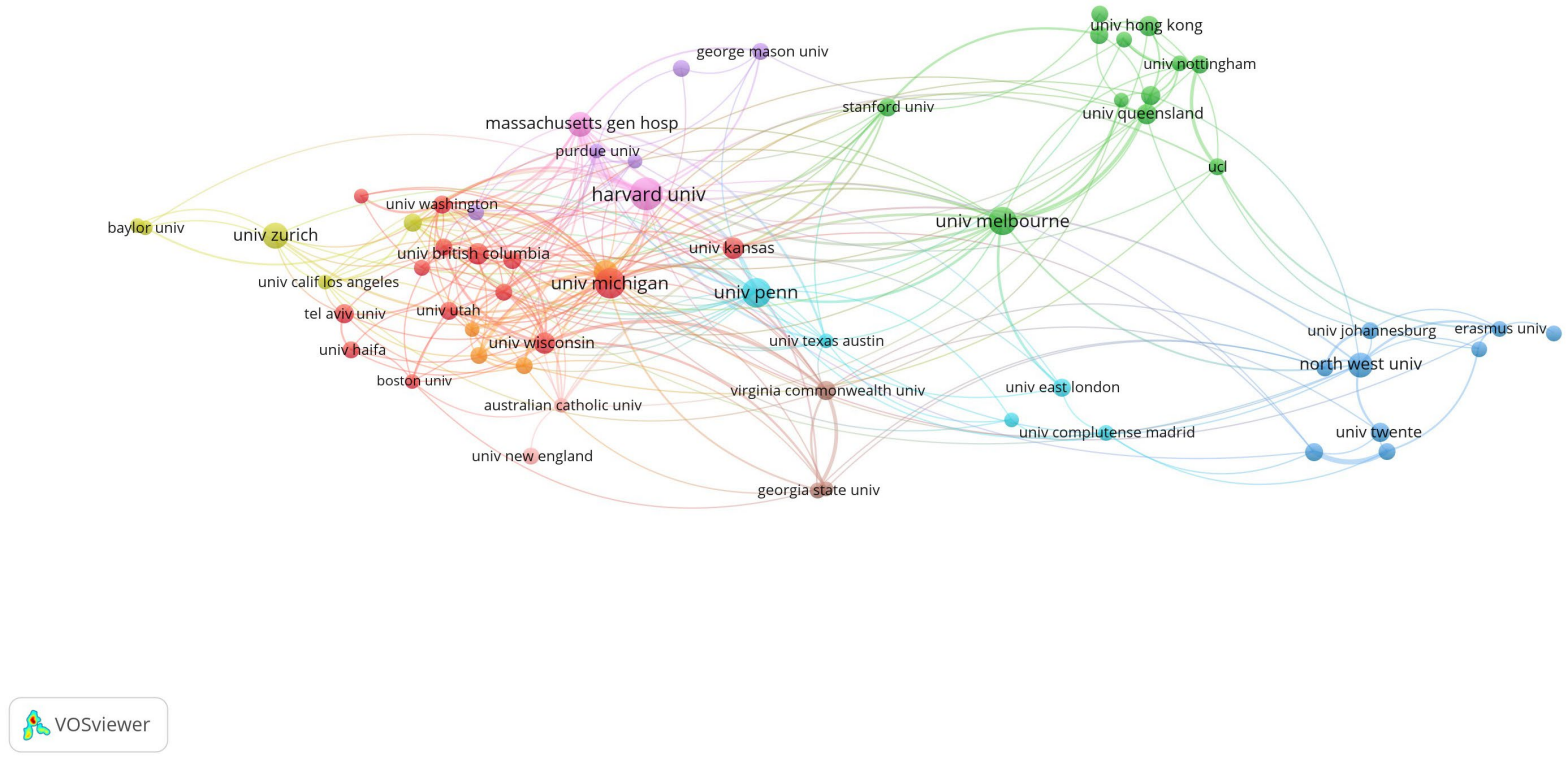
Map of institutions in positive psychology research.

We use the parameters of the number of publications (≥20) and the strength of the line (≥1), generating 61 nodes and 268 links in the network of partner universities (Figure 3). The research institution knowledge map assists us in understanding the key research institutions in this subject as well as their collaborative links. Harvard University, the University of Michigan, the University of Pennsylvania, and the University of Melbourne are all prominently displayed in Figure 3. In terms of the number of publications, 11 universities rank in the top 10. Table 2 shows that each organization participated in at least 43 studies related to positive psychology. Seven of them are from the United States, with the others coming from Australia, Switzerland, South Africa, and Canada. Harvard University was placed first among these universities, with 104 studies completed, followed by the University of Michigan (*n* = 87) and the University of Pennsylvania (*n* = 84). The top 10 institutions, as shown in Table 2, produced 15.50% of all publications. Among these institutions, the University of Michigan had the most WoS citations (13,234) and citations per article (152.11). The top 10 producing institutions are listed in Table 2.

**Table 2:** Top 10 prolific institutions researching positive psychology.

Ranking	Publications	Total link strength	Citations WoS	Citations per paper	Institution	Country
1	104	98	3,385	32.5	Harvard University	USA
2	87	48	13,234	152.11	University of Michigan	USA
3	84	37	11,293	134.44	University of Pennsylvania	USA
4	77	44	2,030	26.36	University of Melbourne	Australia
5	65	10	2,235	34.38	University of Zurich	Swiss
6	60	18	894	14.90	North-West University	South Africa
7	58	76	909	15.67	Massachusetts General Hospital	USA
8	56	44	6,253	111.66	University of North Carolina	USA
9	44	7	1,061	24.11	University of Kansas	USA
10	43	12	1,344	31.26	University of British Columbia	Canada
	43	25	325	7.56	University of Wisconsin	USA

### 3.4. Analysis of journals and co-cited journals

**Figure 4:**
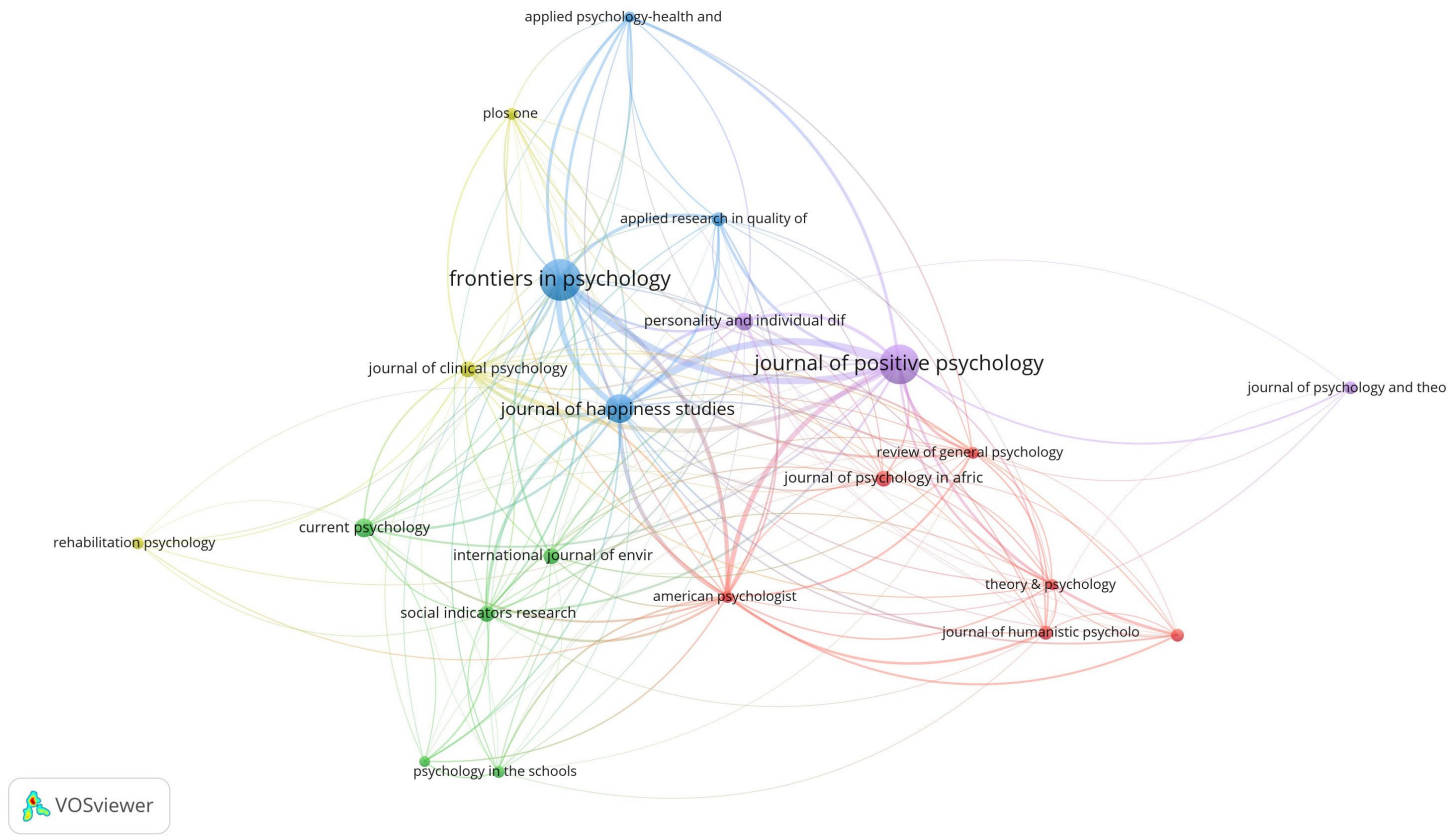
Map of journals in positive psychology research.

In Figure 4, we use the parameters of the number of publications (≥20) and the strength of the line (≥1), generating 21 nodes and 156 links in the journal citation map. The top 10 academic journals that publish articles on positive psychology research are shown in Table 3. These publications varied in IF from 0.917 to 4.614 (average IF: 3.228), and they are specialized journals in this field. Of these, the *International Journal of Environmental Research and Public Health* has the highest factor of influence (4.614). The 10 journals published a total of 986 papers connected to positive psychology research, accounting for 22.52% of the 4,378 studies collected. At least 138 papers were published in the top three journals. In terms of link strength, the *Journal of Positive Psychology* ranked first (*n* = 1,431), followed by the *Journal of Happiness Studies* (*n* = 1,130) and *Frontiers in Psychology* (*n* = 1,096). On this subject, they are quite important.

**Table 3:** Top 10 scholarly journals and co-cited journals in positive psychology research.

Ranking	Publications	IF(Q) (2021)	Journal	Co-citation counts	IF(Q) (2021)	Co-cited journal
1	288	4.232	Frontiers in Psychology	8,469	8.460	Journal of Personality and Social Psychology
2	253	4.290	Journal of Positive Psychology	7,341	16.358	American Psychologist
3	138	4.087	Journal of Happiness Studies	4,057	4.290	Journal of Positive Psychology
4	57	2.387	Current Psychology	3,589	4.087	Journal of Happiness Studies
5	54	3.950	Personality and Individual Differences	3,085	23.027	Psychological Bulletin
6	42	4.614	International Journal of Environmental Research and Public Health	2,783	3.950	Personality and Individual Differences
7	42	2.935	Social Indicators Research	2,215	2.935	Social Indicators Research
8	41	2.995	Journal of Clinical Psychology	2,075	11.802	Journal of Applied Psychology
9	39	0.917	Journal of Psychology in Africa	1,633	2.995	Journal of Clinical Psychology
10	32	1.874	Journal of Humanistic Psychology	1,592	4.232	Frontiers in Psychology

Table 3 displays the top 10 co-cited journals. The journals with the highest academic power and important positions in the field are those with a high co-citation count. In terms of IF, these journals ranged from 2.935 to 23.027 (average IF: 8.214), and they are professional journals in this field. The most influential of these is *Psychological Bulletin* (23.227), followed by *American Psychologist* (16.358) and *Journal of Applied Psychology* (11.802). The *Journal of Personality and Social Psychology* had the highest number of co-citations (8,469), followed by *American Psychologist* (7,341), and then the *Journal of Positive Psychology* (4,057). As a result of the examination of the co-citation count, the *Journal of Personality and Social Psychology* has been recognized as the core journal in the positive psychology research area.

### 3.5. Analysis of authors and co-cited authors

**Figure 5:**
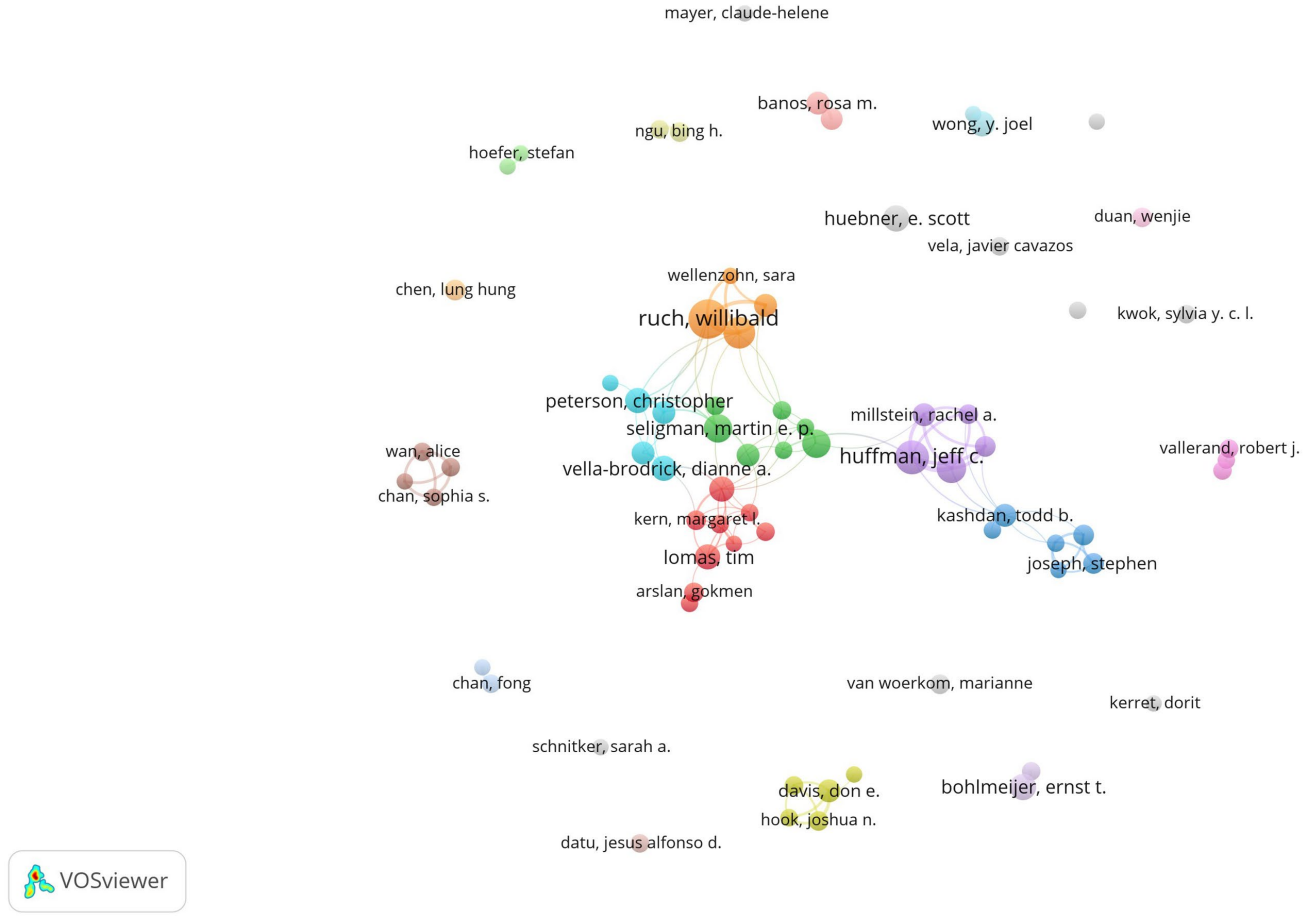
The authors’ map of positive psychology research.

**Figure 6:**
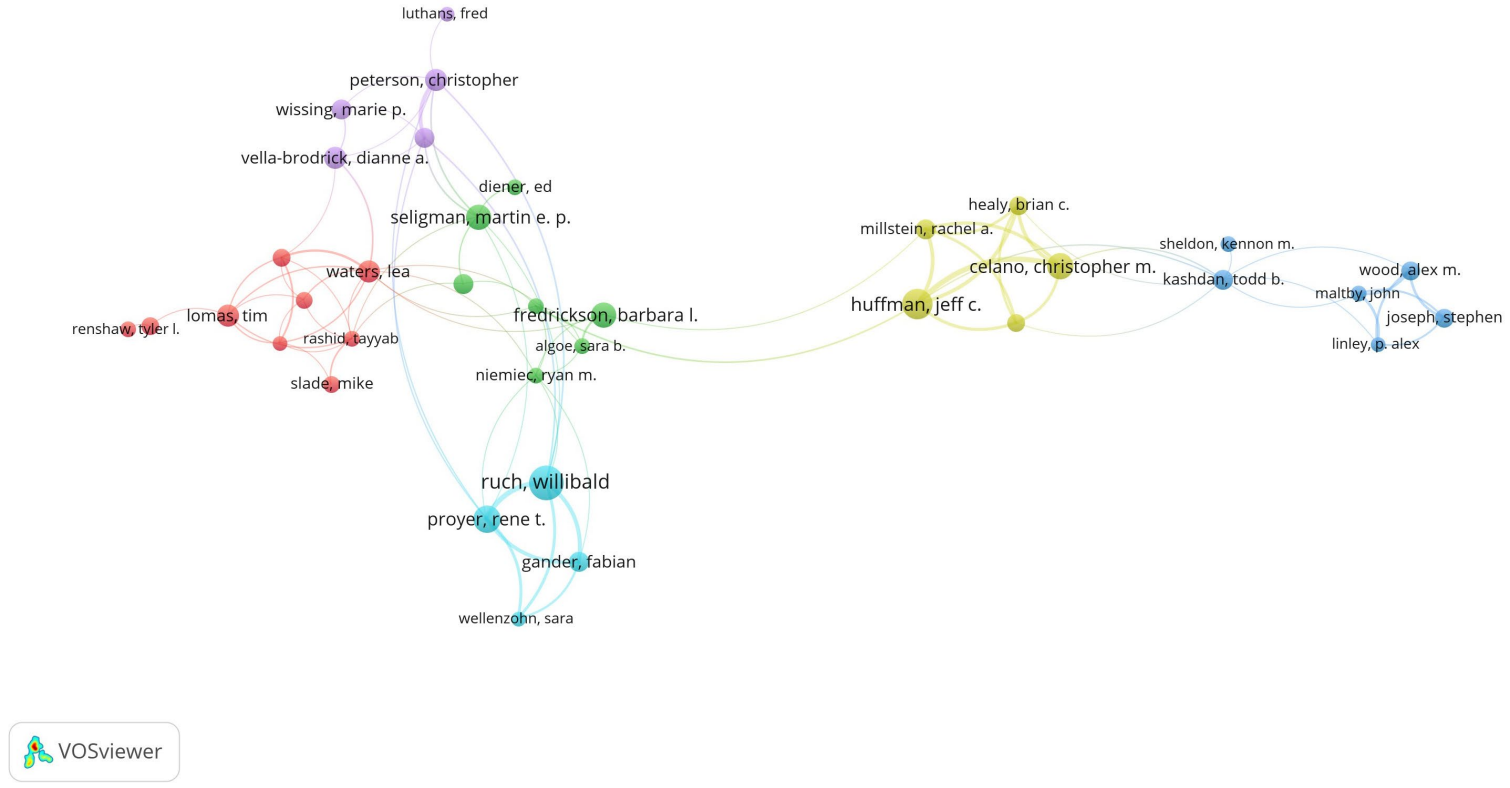
The largest author collaboration network in positive psychology research.

For authors who posted more than 10 publications, generating a co-author map using VOSviewer resulted in 72 nodes and 107 links (Figure 5). The largest network of partnerships we found included Huffman JC, Proyer RT, Fredrickson BL, Ruch W, and other lead authors (Figure 6). In terms of publications, Ruch W published most of the research (51), followed by Huffman JC (40), Celano CM (30), and Proyer RT (30). In terms of citations, the top three authors are Seligman MEP (3,350), Wood AM (3,284), and Maltby J (2,432). Author Maltby J published most of the citations per paper (221.09) in terms of the number of citations per paper, followed by Wood AM (218.93) and Seligman MEP (159.52). Table 4 displays the top 10 authors in terms of publications, citations, and citations per paper in positive psychology research. They are well-known and active authors in this discipline. The top 10 co-cited authors are also shown in Table 4. The most co-cited author is Seligman MEP (5,020), followed by Diener E (2,809) and Fredrickson BL (2,434). These authors have made remarkable contributions to the field of positive psychology.

**Table 4:** Top 10 active authors and co-authors in positive psychology research.

Ranking	Publications	Author	Citations	Author	Citations per paper	Author	Co-citation counts	Cited author
1	51	Ruch W	3,350	Seligman MEP	221.09	Maltby J	5,020	Seligman MEP
2	40	Huffman JC	3,284	Wood AM	218.93	Wood AM	2,809	Diener E
3	30	Celano CM	2,432	Maltby J	159.52	Seligman MEP	2,434	Fredrickson BL
4	30	Proyer RT	2,078	Fredrickson BL	121.90	Diener E	1,734	Peterson C
5	25	Fredrickson BL	1,891	Ruch W	115.07	Joseph S	1,483	Snyder CR
6	23	Lomas T	1,726	Joseph S	94.70	Westerhof GJ	1,389	Lyubomirsky S
7	21	Seligman MEP	1,405	Peterson C	93.00	Rashid T	1,114	Ryff CD
8	20	Peterson C	1,311	Park N	83.12	Fredrickson BL	973	Keyes C
9	20	Wong YJ	1,219	Diener E	77.12	Park N	967	Ryan R
10	18	Davis DE	1,186	Proyer RT	76.08	Vallerand RJ	881	Luthans F
	18	Gander F						
	18	Huebner ES						

### 3.6. Analysis of co-cited references

Essentially, science is a dynamic accumulation process. This means that when scholars write scientific papers, they need to cite others’ academic works (Shafique, 2013). The basis of this field of study is represented by the co-citations, which relate to the references that are also listed in the reference lists of other works. CiteSpace allows for automatic labeling of clustering, greatly reducing the subjectivity of the study of search bounds (Hou et al., 2018).

**Figure 7:**
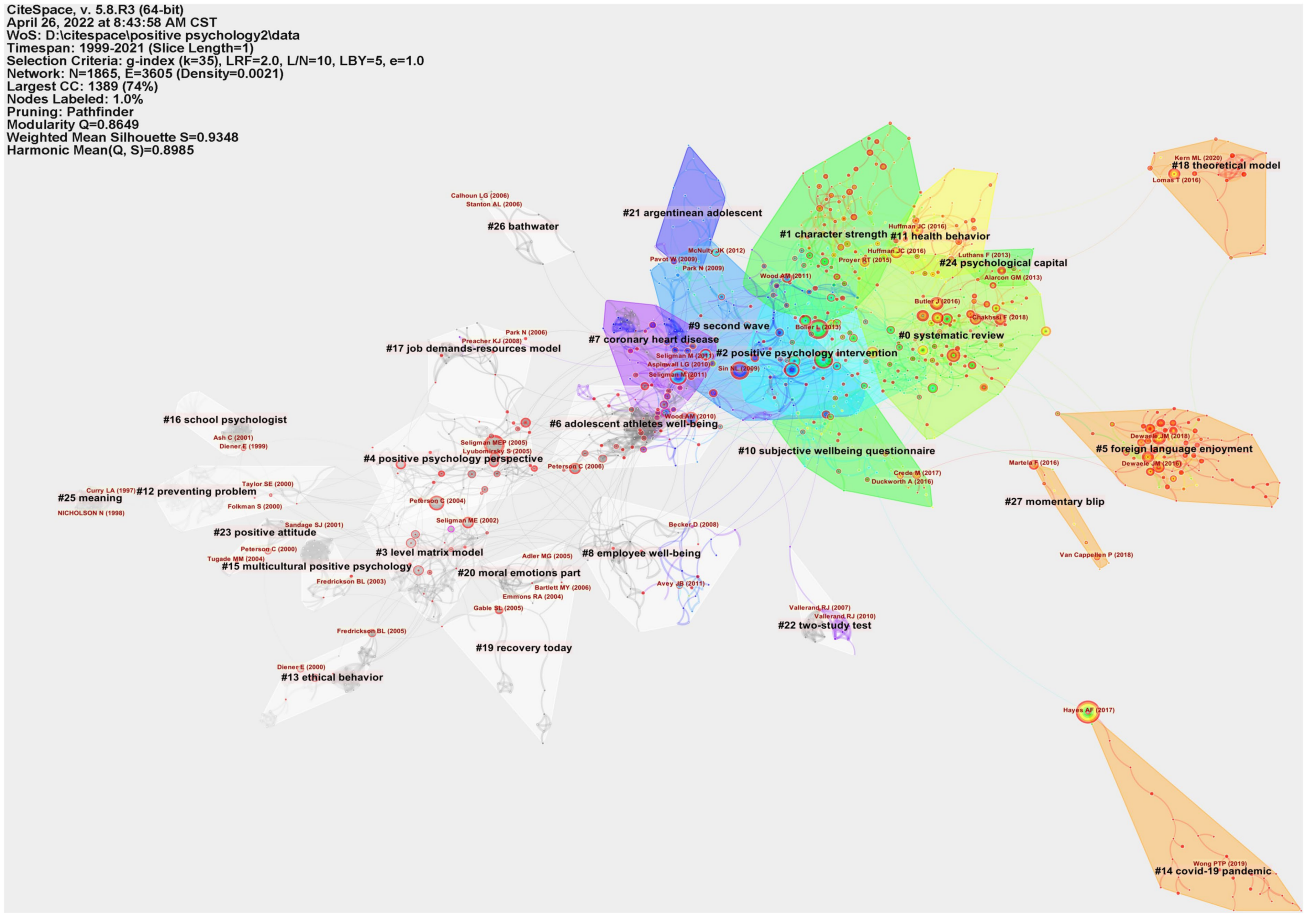
Reference co-citation network analysis of publications in positive psychology research. Cluster visualization of the reference co-citation map.

Figure 7 depicts a cluster visualization of the CiteSpace software-generated coreference network, which was split into 28 clusters, only the 11 largest of which were retrieved from the references based on indexing terms and determined by a log-likelihood ratio algorithm. They are depicted in the image with various convex hulls, including systematic review (cluster #0), character strength (cluster #1), positive psychology intervention (cluster #2), level matrix model (cluster #3), positive psychology perspective (cluster #4), foreign language enjoyment (cluster #5), adolescent athlete well-being (cluster #6), coronary heart disease (cluster #7), employee well-being (cluster #8), the second wave (cluster #9), and subjective wellbeing questionnaire (cluster #10). The authors of each node in the map are identified in red, and each node indicates a referenced reference. The reference co-citation map’s cluster representation is shown in Figure 7. Table 5 displays the characteristics of the top 11 reference clusters in the co-citation network. The configuration’s overall clarity is stronger the closer each cluster’s silhouette score is to one (Chen, 2020). Each cluster had an average silhouette greater than 0.9348 and an overall *Q*-value of 0.8649, indicating that the quality of the cluster was extremely reliable.

**Table 5:** The largest 11 clusters of references in the co-citation network.

Cluster	Size	Mean silhouette	Mean year	Label (LLR algorithm)	Representative reference
0	150	0.872	2014	systematic review	Hendriks et al. (2020)
1	117	0.898	2014	character strength	Waters et al. (2021)
2	104	0.886	2011	Positive psychology intervention	Bolier et al. (2013)
3	83	0.892	2002	level matrix model	Cameron (2004)
4	82	0.913	2003	positive psychology perspective	Wood (2010)
5	79	0.981	2017	foreign language enjoyment	Elahi Shirvan et al. (2021)
6	78	0.978	2007	adolescent athletes well-being	Wood (2010)
7	77	0.914	2009	coronary heart disease	Gana (2013)
8	72	0.914	2007	employee well-being	Luthans (2009)
9	66	0.949	2010	second wave	Wissing (2014)
10	51	0.947	2013	subjective wellbeing questionnaire	Magyar-Moe (2015)

**Figure 8:**
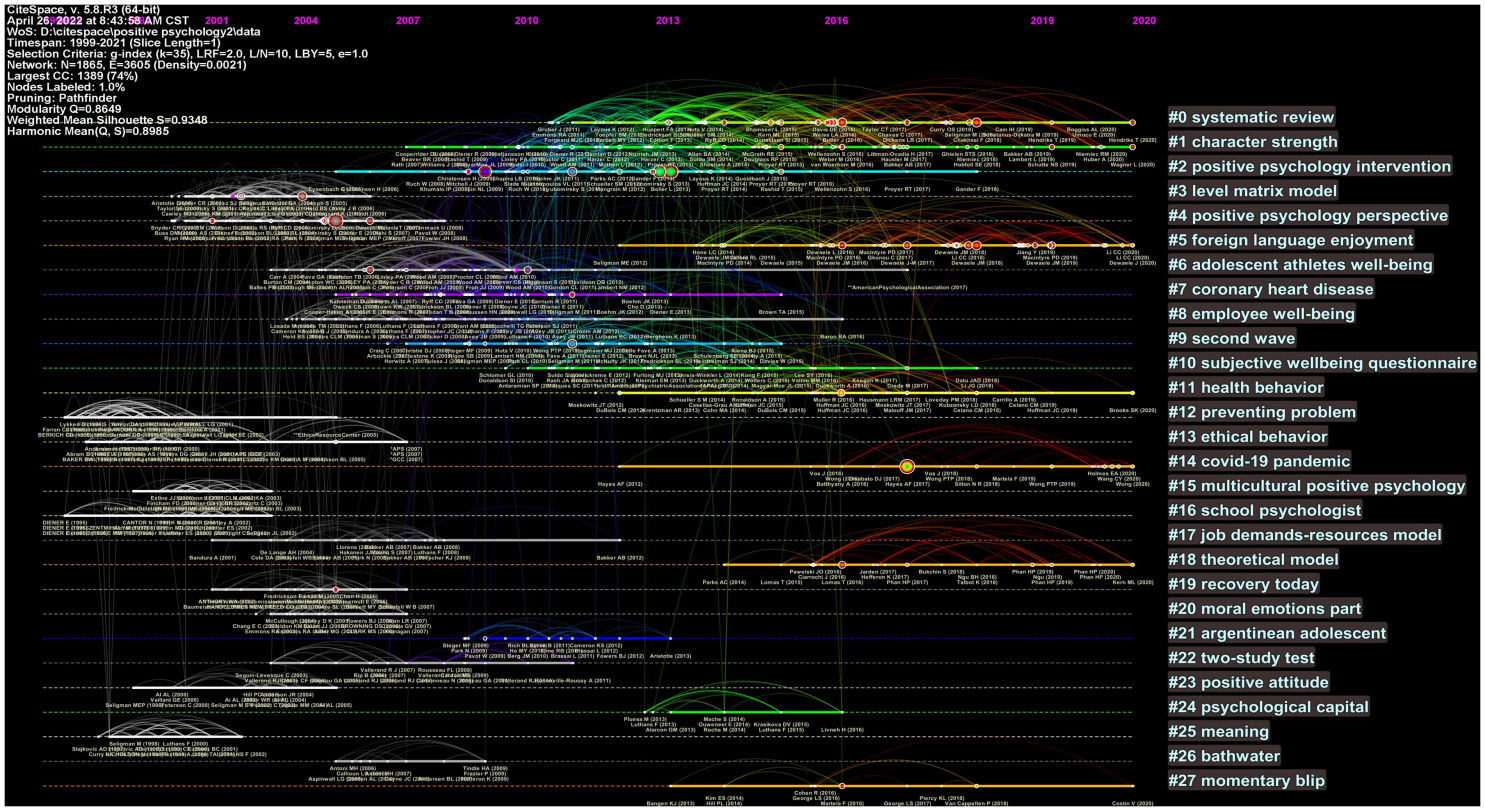
Timeline view in positive psychology research.

We can map the same group of words to the same horizontal axis in the timeline view, with the document located below the horizontal axis; the closer to the left, the newer the document (Figure 8). The figure allows us to see the temporal characteristics of each cluster and the extent of clustering. A total of seven clusters lasting until 2020 are shown in the figure, including systematic review (cluster #0), character strength (cluster #1), foreign language enjoyment (cluster #5), health behavior (cluster #11), COVID-19 pandemic (cluster #14), theoretical model (cluster #18), and momentary blip (cluster #27). Indicating that these fields of research are still receiving scholarly attention.

The top five references in positive psychology research were listed according to their characteristics (see Supplementary material for details). They are regarded as the cornerstone research for the field of positive psychology. The top-ranked paper was published by Seligman MEP, with 1,619 co-citations. The articles with the most co-citations are usually key foundational works in this discipline. *Positive Psychology: an Introduction* received the most co-citations, making it the most important reference. The research concludes by defining the positive psychology scientific framework, pointing out knowledge gaps, and predicting the future of science careers in the twenty-first century. Furthermore, the authors noted that it enables individuals, communities, and society to thrive (Seligman and Csikszentmihalyi, 2000).

**Figure 9:**
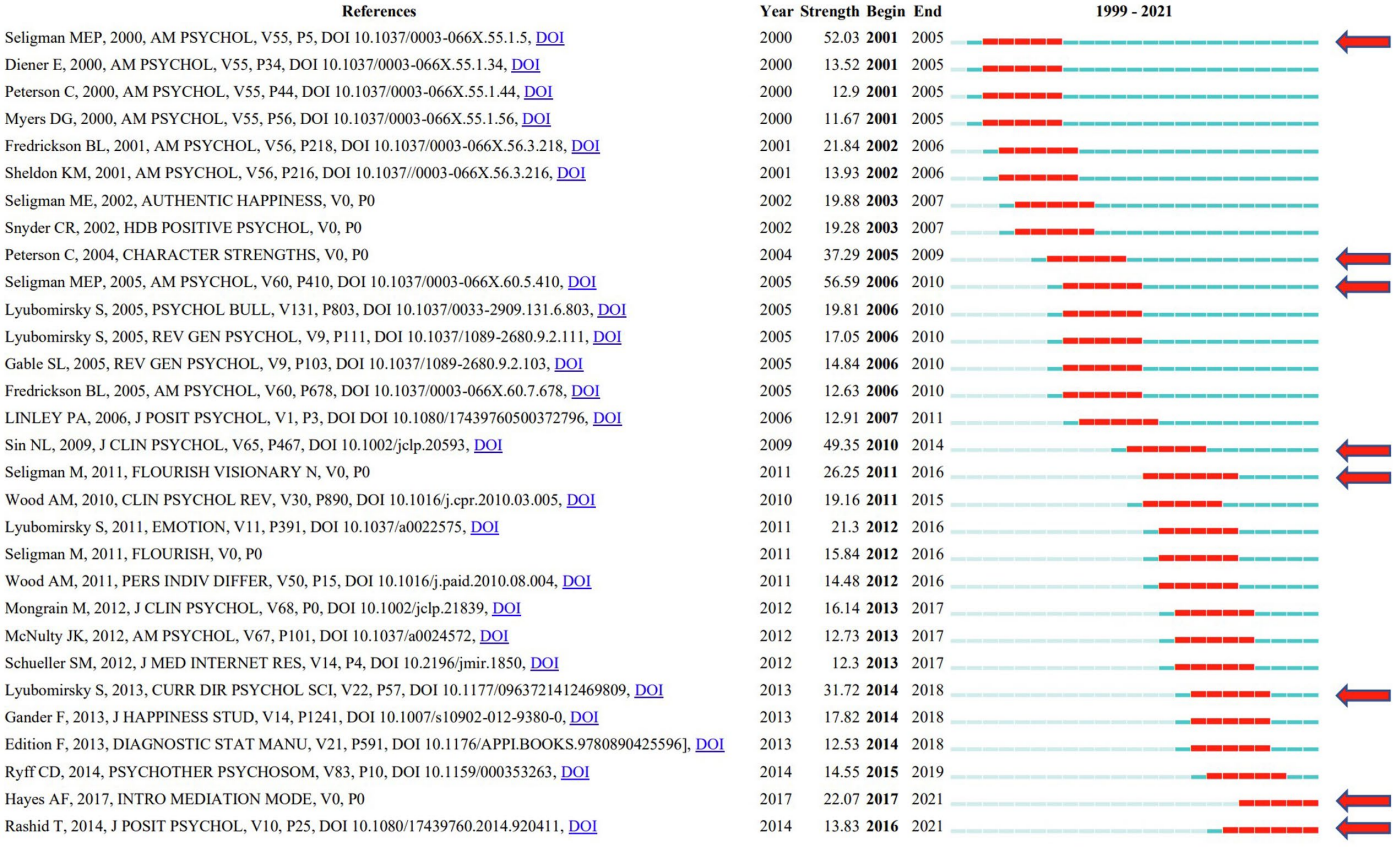
Top 30 references with strong citation bursts in positive psychology research.

While the strongest reference citation burst is considered the primary knowledge of the trend. As can be seen, Seligman MEP led the reference burst in 2000, and the burst was 52.03. The top 30 most potent citation bursts from 1999 through 2021 are shown in Figure 9. Additionally, the number of red squares corresponds to the time of the epidemic in the literature, and each red square indicates a year. Figure 9 lists a few important works of literature. It demonstrates that the red arrow’s target reference is a crucial one with a powerful explosion. Seligman and Csikszentmihalyi (2000), Peterson (2004), Seligman et al. (2005), Sin and Lyubomirsky (2009), Seligman (2011), and Lyubomirsky and Layous (2013) are a few examples of references. Hayes (2017) and Rashid (2014) are still bursting and need our high attention.

Important references are also included in Table 6. Bolier et al. (2013) is the highest-ranked object per burst in Cluster #2, with bursts of 57.41. The second is Seligman et al. (2005) in Cluster #4, with bursts of 56.59. The third is Seligman and Csikszentmihalyi (2000) in Cluster #248, with bursts of 52.03. The 4th is Sin and Lyubomirsky (2009) in Cluster #2, with bursts of 49.35. Peterson (2004) is the 5th in Cluster #3, with a burst number of 37.29. More detailed information is shown in Table 6.

**Table 6:** Top 10 co-cited references in positive psychology research in terms of citation bursts.

Bursts	Reference	DOI	Cluster-ID
57.41	Bolier et al. (2013)	10.1186/1471-2,458-13-119	2
56.59	Seligman et al. (2005)	10.1037/0003-066X.60.5.410	4
52.03	Seligman and Csikszentmihalyi (2000)	10.1037/0003-066X.55.1.5	248
49.35	Sin and Lyubomirsky (2009)	10.1002/jclp.20593	2
37.29	Peterson (2004)		3
31.72	Lyubomirsky and Layous (2013)	10.1177/0963721412469809	2
26.25	Seligman (2011)		9
22.07	Hayes (2017)		14
21.84	Fredrickson (2001)	10.1037/0003-066X.56.3.218	4
21.30	Lyubomirsky (2011)	10.1037/a0022575	2

## 4. Discussion

### 4.1. General information in positive psychology research

In this study, a bibliometric analysis of positive psychology research was conducted from 1999 to 2021. Based on the overall analysis of publications, productive countries/regions, institutions, journals, and authors, we can provide further research suggestions for researchers. The summary is as follows:

- Publications in the field of positive psychology have continued to grow since 1999. The annual output of positive psychology publications from 1999 to 2021 was divided into three phases. Less than 100 publications were generated annually during the first phase, which lasted from 1999 to 2007. The second phase lasted from 2008 until 2018. The number of publications has been steadily increasing. For the first time, more than 100 papers were published in 2008, and that number climbed to 378 articles in 2018. The third phase lasted from 2019 to 2021. In the past 3 years, there has been a considerable growth in the number of articles published; all of them have exceeded 500 articles. In general, positive psychology is still a popular area of study.
- With global communication, the physical distance between collaborators in research becomes increasingly irrelevant (Ruhl and Priede, 2011), and researchers in the field of positive psychology are also engaged in extended cooperation (Figures 2, 3). Based on the number of publications, positive psychology publications from the United States are more influential than those from other countries. Seven of the top 10 institutions are from the United States, which demonstrates that American institutions have significant influence in this field. In addition, in a model for developing countries, China ranked fourth with 361 studies published, and South Africa ranked eighth with 158.
- Analysis of co-cited journals and journals has shown that scholars may focus on all three of these journals when publishing papers, namely, *Frontiers in Psychology* (288), *Journal of Positive Psychology* (253), and *Journal of Happiness Studies* (138). And when referring to articles, scholars can focus on these journals, for example, the *Journal of Personality and Social Psychology* (8,469), *American Psychologist* (7,341), *Journal of Positive Psychology* (4,057), *Journal of Happiness Studies* (3,589), and *Psychological Bulletin* (3,085).
- In the co-author map, Ruch has the most publications, Seligman has the most citations, and Maltby J has the most citations per paper. These three authors are thus important influences in positive psychology research. The most co-cited author is Seligman (5,020), followed by Diener (2,809) and Fredrickson (2,434). Seligman is a well-known and extensively quoted author on positive psychology. In 1999, he released an essay describing the significance of positive psychology research (Gillham and Seligman, 1999), which also served as the inception of the discipline.

### 4.2. Emerging trends and hotpots in positive psychology research

The co-cited references are what make up the knowledge base. A key component of CiteSpace is co-citation analysis. The time slice used in this study was 1 year; the selection criteria used a modified g index (*k* = 35); and the period is from 1999 to 2021 years. CiteSpace describes the trends and patterns of change in the co-citation reference map, which can be used to capture the research focus of the prospective scientific community. The node in Figure 7 represents a single reference, and the line indicates that the two references are connected in some way.

The most representative articles of the cluster list in Supplementary material. For example, in cluster #0, the papers cited that are most related to the cluster are Hendriks et al. (2020), which cited 19% of the contributions of the cluster; Neumeier et al. (2017), Moskowitz et al. (2020), Hausler et al. (2017), which cited 17%; and Job and Williams (2020), which cited 14% of the literature.

#### 4.2.1. Clustering visualization of the reference co-citation map

CiteSpace divides the co-citation network into clusters of many co-citation references so that connections across clusters are weak but strong inside each cluster. The 11 major clusters are listed in Table 5 according to their size, or the total number of persons in each cluster. Large-membership clusters are displayed. If the silhouette score is close to 1, it indicates that the cluster has better homogeneity or coherence. Table 5 shows that all clusters had a high silhouette score, indicating better homogeneity or coherence. Based on the labels selected for the clusters by the log-likelihood ratio test method (LLR) (Chen et al., 2010). The three largest clusters were analyzed, and the results are as follows:

With 150 members and a silhouette value of 0.872, cluster #0 is the biggest cluster. All references were across 14 years, from 2007 to 2020, and the median year was 2014. It is labeled “systematic review” by LLR. Hendriks et al. (2020) published an article that cited the most references in cluster #0. Through a detailed review and meta-analysis, this paper aims to determine if MPPIs are effective. These findings show that MPPI is successful in enhancing mental health. Further good research in different populations is needed to strengthen the claim for the effectiveness of MPPI. While the label for this cluster is “systematic review,” which states that the article type is “review,” the focus of the article is primarily on positive psychology interventions. Neumeier et al. recently developed an online intervention program aimed at improving employees’ well-being (Neumeier et al., 2017). Moskowitz et al. reviewed emotion measurement in positive psychology interventions (Moskowitz et al., 2020), and Job and Williams reviewed the role of online positive psychology interventions in sexual and gender minorities (SGM) (Job and Williams, 2020). From many reviews, it is also found that there has been a lot of research on positive psychology intervention, which is a hot spot in the field of positive psychology.

With 117 members and a silhouette value of 0.898, cluster #1 is the second-largest cluster. For all references across 10 years (from 2011 to 2020), the median year was 2014. It is labeled as a character strength by LLR. Waters et al. (2021) published an article that cited the most references in cluster #1. The authors underline that strengthening mental health during COVID-2019 and developing positive processes and capacities will benefit the future development of mental health. This study covers the research and applied positive psychology themes of meaning, coping, self-compassion, courage, gratitude, personality advantage, positive emotion, a positive interpersonal process, and high-quality connection to help individuals cope with the epidemic. Apart from this paper, other papers also satisfy the clustering theme of “character strength.” Miglianico et al. (2019) review the literature on the use and development of strengths in the workplace. Strecker et al. (2020) will discover the circumstances for the use of individual character strengths in the workplace, resulting in enhanced job engagement and well-being. Mayerson (2020) proposed a model for the role of character strengths in the success of individuals, groups, and species. Hausler et al. (2017) examined the individual relationships between 24 different aspects of personality strengths, subjective well-being (SWB), and psychological well-being (PWB). Overall, the correlation between “good personality” and PWB was significantly stronger than that of SWB. As can be seen in Cluster #1, not only is “character strength” research emphasized, but more researchers are paying attention to “character strength” in the workplace.

With 104 members and a silhouette value of 0.886, cluster #2 is the third-largest cluster. All references were across 14 years, from 2005 to 2018, and the median year was 2011. It is labeled as a positive psychology intervention by LLR. Bolier et al. (2013) published an article that cited the most references in cluster #2. The goal of this meta-analysis is to look into the effectiveness of a positive psychology intervention on the general population as well as on those who have specific psychosocial issues. Overall, the paper shows that positive psychology interventions can successfully improve subjective and psychological well-being while also assisting in the reduction of depressive symptoms. Figure 7 shows that clusters #2 and #0 partially overlap, there are some similarities between the two clusters, and there are many papers cited to investigate positive psychology intervention. Schueller and Parks (2014) summarized the current state of positive psychology interventions as they relate to self-help and that the next stage in research necessitates the application of these tactics in ways that allow them to be used in real-world circumstances. Gander et al. (2012) conclude that some “strengths-based” therapies can improve happiness. Likewise, Proyer et al. (2014) conducted a positive psychology intervention on older people over 50 years of age, and the results showed that the intervention was effective. The results of the Hone et al. (2014) meta-analysis show that positive psychology interventions have a clear effect on promoting well-being. To maximize the potential of PPI to promote population health, there is a need to extend the efficacy trial report in the future. Positive psychology intervention is a hotspot for positive psychology research. A great deal of research has been conducted by researchers from the perspectives of meta-analysis, literature reviews, and intervention experiments.

#### 4.2.2. Co-citation clusters timeline map

Figure 8 depicts the age span of the literature in each cluster. The clusters are placed vertically in decreasing size order, and each cluster is presented from left to right (Chen, 2017). Based on the timeline map, we should focus on larger and more recent clusters. The three largest clusters are #0, #1, and #2, which were analyzed earlier in this paper. Clusters #5, #14, #18, and #27 are relatively recent in terms of time and require further attention. Since clusters #18 and #27 contain few articles and are not representative, we retain only clusters #5 and #14 for the analysis. The results are as follows:

Cluster #5 consists of 79 members with a silhouette value of 0.981. All references covered over 9 years, from 2012 to 2020, and the median year was 2017. It is labeled “foreign language enjoyment” by LLR. Elahi Shirvan et al. (2021) published an article that cited the most references in cluster #5. In response to the dynamic change in the SLA domain and the necessity for the creation of appropriate methodologies to evaluate the dynamics of developing notions in the field such as grit and pleasure, the current study sought to investigate the rise of foreign language enjoyment (FLE) and L2 grit over time. Wang X. et al. (2021) investigate Chinese university students’ enjoyment of a web-based language learning environment. In L2 education, Li and Xu (2019) found that an intervention focused on emotional intelligence has a good effect on promoting positive emotions. At the same time, as a result of the positive impact of second language acquisition (SLA) on the promotion of academic achievement and language learners’ well-being (Guo, 2021). Recently, Wang Y. L. et al., (2021) reviewed the role of positive psychology in promoting second language learning. Accordingly, until recently, with the emergence and rapid development of positive psychology in general education (Dewaele, 2014), there has been a clear positive revival in the area of L2 education (Lake, 2015; Kruk, 2019), which has also emerged as a hotspot for study and a trend in the discipline of positive psychology.

Cluster #14 consists of 31 members and has a silhouette value of 1. This cluster has the highest homogeneity or coherence, indicating that the degree of coherence in the literature in this cluster is the highest. All references were over 9 years from, 2012 to 2020, and the median year was 2018. It is labeled as a COVID-19 pandemic by LLR. Waters et al. (2021) published an article that cited the most references in cluster #14. Although almost all of the papers cited within the cluster were published recently, the topics covered were COVID-19 and positive psychology, demonstrating that positive psychology plays a crucial role in the epidemic (Quiroga-Garza et al., 2021). The COVID-19 epidemic had a significant impact on people’s lives and mental health (Luo et al., 2020). Researchers should concentrate more on applying positive psychology to COVID-19.

## 5. Conclusion

In conclusion, our bibliometric analysis of positive psychology found that positive psychology is a rapidly growing discipline with some achievements that warrant further research. In this study, Microsoft Excel 2013, VOS viewer (1.6.17), and CiteSpace (5.8.R1) software were used to analyze the annual number of documents, cooperation networks (countries/regions, institutions, journals and cited journals, authors and cited authors), and total cited documents. By analyzing data from the large-scale literature, we can gain a comprehensive understanding of the development of the field of positive psychology research and the research trends in the field.

We can understand the general information in this field. Firstly, the number of papers published on this topic continues to grow, indicating that it is a research hotspot in the field of psychology. Secondly, in the analysis of the cooperation network, we can find that the United States and the institutions of the United States occupy a dominant position in this field; in journals, we should pay attention to several major journals, such as *Frontiers in Psychology*, *Journal of Positive Psychology*, *Journal of Happiness Studies*, and so on; In terms of an author analysis, authors such as Ruch W, Huffman JC, Celano CM, Proyer RT, Fredrickson BL have more output, while authors such as Seligman MEP, Diener, ED, Fredrickson BL, Peterson C, Snyder CR have been cited more and have a greater impact.

Analysis of the cited literature allows us to understand the research base and research frontier in this field. First, jointly cited literature forms the research base for a research field. Documents such as Bolier et al. (2013), Seligman et al. (2005), Seligman and Csikszentmihalyi (2000), Sin and Lyubomirsky (2009), and Peterson (2004) can be found to be highly explosive in nature, indicating that these papers are the foundation for the development of this field, to which we must pay close attention. Second, through cluster analysis of co-citations, we can find research hotspots and development trends in this field. The systematic review, character strengths, positive psychology intervention, language enjoyment, and the COVID-19 pandemic are the foci of research and developmental trends in this field that need our high attention.

## 6. Strengths and limitations

This is the first large-scale data analysis of positive psychology papers utilizing CiteSpace and VOSviewer software. Furthermore, our findings offer a clear visual analysis, and so forth, of positive psychology publications. In addition, the co-citation analysis can also capture the research base and hot trends in this field, providing a reference for researchers to fully understand this field.

This study used the scientometric method for literature analysis, which has objectivity but also some limitations. First, the results of the software analysis are somewhat mechanical and require us to select meaningful results. At the same time, there is a possibility of ignoring some meaningful literature. For example, in considering the role of positive psychology in global issues, some researchers suggest that positive psychology may benefit from the integration of spirituality to better support people’s well-being (Bellehumeur et al., 2022); others have found that positive psychology has a considerable impact on employees’ green behavior (Meyers and Rutjens, 2022). Second, we did not conduct an in-depth assessment of the literature, only those in WoSCC; other databases, such as Scopus, MEDLINE, and PubMed, are available. We analyzed the type of literature and selected only papers and reviews, ignoring other types of literature. We also analyzed only English literature and ignored literature in other languages, which may have led to a lack of attention to other cultures. Related to this is the need to recognize the parochial nature of positive psychology, which seems to be US-centric, especially in terms of leadership (Ryff, 2022). All of these may have biased the data. For example, in studies across cultures, Appiah et al. (2020) found that positive psychology interventions promote mental health among rural Ghanaian adults; a study in Hong Kong, China, found that a multifaceted positive psychology program was effective in reducing adolescent anxiety and increasing subjective well-being (Kwok et al., 2022). Finally, potential bias in the data may be caused by duplicate author names of authors, or the use of different names by the same author; or by irregularities in literature citation, where different authors cite the same literature in different formats in the analysis of co-cited literature. This is where the practice of some researchers is worthy of consideration; for example, Donaldson et al. (2014) coded all articles in their review by raters using a systematic coding scheme.

## Data availability statement

The raw data supporting the conclusions of this article will be made available by the authors, without undue reservation.

## Author contributions

FW and JG contributed to the study design, acquisition of research data, and drafted the manuscript. FW conducted the data analysis. GY contributed to critical revising of the manuscript. All authors contributed to the article and approved the submitted version.

## Funding

This study was supported by the National Social Science Fund Project of China (No. 19XSH018).

## Conflict of interest

The authors declare that the research was conducted in the absence of any commercial or financial relationships that could be construed as a potential conflict of interest.

## Publisher’s note

All claims expressed in this article are solely those of the authors and do not necessarily represent those of their affiliated organizations, or those of the publisher, the editors and the reviewers. Any product that may be evaluated in this article, or claim that may be made by its manufacturer, is not guaranteed or endorsed by the publisher.
