# Supplementary material for: Study on positive psychology from 1999 to 2021: A bibliometric analysis
Source: Front Psychol. 2023 Mar 1;14:1101157. doi: 10.3389/fpsyg.2023.1101157 (PMC10015893; doi:10.3389/fpsyg.2023.1101157)
Supplement: Supplementary file 1 [file Data_Sheet_1.docx]

**Supplementary Material**

**Table 7** | Top five co-cited references in positive psychology research.

| Ranking | Co-citation counts | Cited reference | Journal | Main finding |
| --- | --- | --- | --- | --- |
| 1 | 1619 | Positive psychology - An introduction (Seligman and Csikszentmihalyi, 2000) | American Psychologist | The authors outline a framework for a science of positive psychology, point to gaps in our knowledge, and predict that the next century will see a science and profession that will come to understand and build the factors that allow individuals, communities, and societies to flourish. |
| 2 | 866 | Positive Psychology Progress: Empirical Validation of Interventions (Seligman et al., 2005) | American Psychologist | Positive interventions can supplement traditional interventions that relieve suffering and may someday be the practical legacy of positive psychology. |
| 3 | 821 | The role of positive emotions in positive psychology - The broaden-and-build theory of positive emotions (Fredrickson, 2001) | American Psychologist | The theory and findings suggest that the capacity to experience positive emotions may be a fundamental human strength central to the study of human flourishing. |
| 4 | 598 | The Satisfaction With Life Scale (Diener et al., 1985) | Journal of Personality Assessment | This article reports the development and validation of a scale to measure global life satisfaction, the Satisfaction With Life Scale (SWLS). |
| 5 | 544 | Enhancing Well-Being and Alleviating Depressive Symptoms With Positive Psychology Interventions: A Practice-Friendly Meta-Analysis (Sin and Lyubomirsky, 2009) | Journal of Clinical Psychology | Clinicians should be encouraged to incorporate positive psychology techniques into their clinical work, particularly for treating clients who are depressed, relatively older, or highly motivated to improve. Our findings also suggest that clinicians would do well to deliver positive psychology interventions as individual (versus group) therapy and for relatively long periods. |

**Table 8** | Cited references and citing articles of Cluster #0, #1, #2, #5, #14

|  | Citing articles | |
| --- | --- | --- |
|  | Coverage % | Title (Author, Year) |
| Cluster #0 systematic review | 19 | The Efficacy of Multi-component Positive Psychology Interventions: A Systematic Review and Meta-analysis of Randomized Controlled Trials (Hendriks et al., 2020) |
|  | 17 | Delivering your daily dose of well-being to the workplace: a randomized controlled trial of an online well-being programme for employees (Neumeier et al., 2017) |
|  | 17 | Measuring Positive Emotion Outcomes in Positive Psychology Interventions: A Literature Review (Moskowitz et al., 2020) |
|  | 17 | Distinguishing Relational Aspects of Character Strengths with Subjective and Psychological Well-being (Hausler et al., 2017) |
|  | 14 | Translating Online Positive Psychology Interventions to Sexual and Gender Minorities: A Systematic Review (Job and Williams, 2020) |
| Cluster #1 character strength | 16 | Positive psychology in a pandemic: buffering, bolstering, and building mental health (Waters et al., 2021) |
|  | 15 | Strength Use in the Workplace: A Literature Review (Miglianico et al., 2019) |
|  | 15 | Identifying thriving Workplaces in Hospitals: Work Characteristics and the Applicability of Character Strengths at Work (Strecker et al., 2020) |
|  | 15 | The Character Strengths Response: An Urgent Call to Action (Mayerson, 2020) |
|  | 14 | Distinguishing Relational Aspects of Character Strengths with Subjective and Psychological Well-being (Hausler et al., 2017) |
| Cluster #2 positive psychology intervention | 18 | Positive psychology interventions: a meta-analysis of randomized controlled studies (Bolier et al., 2013) |
|  | 18 | The science of self-help translating positive psychology research into increased individual happiness (Schueller and Parks, 2014) |
|  | 14 | Strength-Based Positive Interventions: Further Evidence for Their Potential in Enhancing Well-Being and Alleviating Depression (Gander et al., 2012) |
|  | 13 | Positive psychotherapy: A strength-based approach (Rashid, 2014) |
|  | 13 | Positive psychology interventions in people aged 50-79 years: long-term effects of placebo-controlled online interventions on well-being and depression (Proyer et al., 2014) |
|  | 13 | An evaluation of positive psychology intervention effectiveness trials using the re-aim framework: A practice-friendly review (Hone et al., 2014) |
| Cluster #5 foreign language enjoyment | 24 | A Longitudinal Study of Foreign Language Enjoyment and L2 Grit: A Latent Growth Curve Modeling (Elahi Shirvan et al., 2021) |
|  | 22 | Investigating Chinese University Students’ Enjoyment in a Web-Based Language Learning Environment: Validation of the Online Foreign Language Enjoyment Scale (Wang et al., 2021a) |
|  | 22 | Trait Emotional Intelligence and Classroom Emotions: A Positive Psychology Investigation and Intervention Among Chinese EFL Learners (Li and Xu, 2019) |
|  | 22 | Exploring the Dynamic Interplay Between Foreign Language Enjoyment and Learner Engagement With Regard to EFL Achievement and Absenteeism: A Sequential Mixed Methods Study (Guo, 2021) |
|  | 22 | Researching and Practicing Positive Psychology in Second/Foreign Language Learning and Teaching: The Past, Current Status and Future Directions (Wang et al., 2021b) |
| Cluster #14 COVID-19 pandemic | 12 | Positive psychology in a pandemic: buffering, bolstering, and building mental health (Waters et al., 2021) |
|  | 10 | How Having a Clear Why Can Help Us Cope With Almost Anything: Meaningful Well-Being and the COVID-19 Pandemic in Mexico (Quiroga-Garza et al., 2021) |
|  | 9 | Meaning-Centered Coping in the Era of COVID-19: Direct and Moderating Effects on Depression, Anxiety, and Stress (Eisenbeck et al., 2021) |
|  | 8 | Tragic Optimism as a Buffer Against COVID-19 Suffering and the Psychometric Properties of a Brief Version of the Life Attitudes Scale (Leung et al., 2021) |
